# Trends in new active tuberculosis incidence in a lower middle income region

**DOI:** 10.1186/1471-2334-13-S1-P89

**Published:** 2013-12-16

**Authors:** Daniela Vişan, Veronica Gogoneață

**Affiliations:** 1 Clinical Hospital Calafat, Romania

## Background

Even though new cases of tuberculosis (TB) have been fewer in the last years and the TB mortality rate has decreased, the global burden of TB remains enormous. One of the major problems is the relation between active TB and underlying diseases. At this moment, the proportion of tuberculosis developed in compromised hosts is still high. Our study aims to evaluate the incidence of and risk factors for new cases of TB in the Calafat region.

## Methods

We performed our study in the TB laboratory unit Calafat, between September 2012 and August 2013.

## Results

The entire population of the Calafat region is estimated at 70,558 inhabitants. During the follow-up period, we diagnosed 55 patients with new active TB infection. Incidence rates were higher for patients: young- average 39.54 (14-81 years old), men- (39 men, 72% vs. 16 women, 28%), from the rural areas- 64% vs. 36%. Two men and three women developed active TB on underlying chronic diseases with immune incompetence- three malignancies (lung, age 67; prostate, age 71; non Hodgkin lymphoma, age 22), one rheumatoid arthritis (age 38) and one HIV infection (age 23).

## Conclusion

TB incidence rate in rural areas is much higher than in the urban areas due to serial socioeconomic problems of the population living there. The presented data suggests that tuberculosis-controlling efforts should be intensified in the rural area.

